# Redefining the Mode of HIV Transmission through Analysis of Risk Attribution among the Reported HIV Cases from 1993 to 2021 in Bhutan

**DOI:** 10.1155/2022/2137164

**Published:** 2022-07-09

**Authors:** Lekey Khandu, Ngawang Choida, Jurmi Drukpa, Dolley Tsehring, Sonam Wangdi

**Affiliations:** ^1^National HIV, Hepatitis and STIs Control Program, Department of Public Health, Ministry of Health, Thimphu, Bhutan; ^2^Health Information and Service Center, Ministry of Health, Thimphu, Bhutan; ^3^Community Health Department, Jigme Dorji National Referral Hospital, Thimphu, Bhutan; ^4^Policy and Planning Division, Ministry of Health, Thimphu, Bhutan

## Abstract

**Introduction:**

The detailed risk assessment of the diagnosed HIV cases in Bhutan is an opportunity to carry out the risk analysis to generate evidence of wherein, under what context, and in which population groups most new infections are occurring. The evidence collected will help to test the current assumption of Bhutan experiencing diffused and generalized HIV epidemic.

**Methods:**

This is a cross-sectional study using a quantitative method to assess the risk behavior of the diagnosed HIV cases from 1993 to 2019. The study also included secondary data analysis of those cases already captured by the routine case-based surveillance from 2020 to 2021. The data collection was done from 1 to 30 January 2022 in all the twenty districts of Bhutan. Descriptive statistical analysis was used to analyze the characteristics of the study population, and relationships were established using the Chi-square Test. We have sought ethics approval and obtained participants' informed consent.

**Results:**

The risk attribution analysis showed that 81.94% of HIV infection among the reported HIV cases in Bhutan has occurred through high-risk heterosexuals and 8.88% through mother-to-child transmission, and parenteral transmission accounts for 1.58% and then 1.35% through homosexual. Of the 81.94% high-risk heterosexuals, 41.08% acquired through sex workers and clients of sex workers, 27.99% from HIV-infected persons, 12.64% from sex work, and 0.23% from injecting drug use.

**Conclusions:**

This study has shed some light on a gradual epidemic shift from the current perceived diffused and generalized to the concentrated epidemic among subpopulation groups like female sex workers and their clients.

## 1. Introduction

Bhutan is one of the few countries in South Asia that continues to experience a low adult (15–49 years) HIV prevalence, estimated to be under 0.2% based on the UNAIDS Spectrum estimation, 2020 [1]. Bhutan has experienced a low-level and diffuse HIV epidemic [2–4]. Compared to other counties in the South and Southeast Asia region, Bhutan's HIV epidemic started later, with the first case diagnosed in 1993, and progressed more slowly. Sporadic cases appeared between 1993 and 2000. From 2000 to 2013, the annual number of new HIV diagnoses rose from 9 to 51. Since 2013, there has been a plateau in the number of new HIV diagnoses, fluctuating between 49 and 58 annually [4,5]. By June 2022, there were a cumulated 835 (433 males and 402 females) HIV diagnoses, 628 of whom are known to be alive, and 608 (96.8%) of whom are on antiretroviral treatment [6]. The majority (70%) of reported HIV cases in Bhutan were between the ages of 25 and 49 years old [6,7], showing that HIV has primarily affected the most economically productive age. The vast majority (94.2%) of cases acquired HIV infection through the sexual route, 5% from mother-to-child transmission (MTCT), and 0.8% from injecting drug use [6]. UNAIDS models place the number of people living with HIV in Bhutan at 1,300 [8] indicating that only 61% of cases have been diagnosed [6]. The national response to HIV aims to end the epidemic by 2030.

Since the detection of the first HIV case in 1993, HIV has spread throughout the country. In the absence of a proper routine case-based surveillance in the country to understand the risk behaviors of newly diagnosed HIV cases, it was assumed that Bhutan is experiencing a diffused and generalized epidemic [9]. As a result, the country has defined HIV-related risk behavior among the reported cases based on the nature of occupation where the highest rates of infection were accorded among the housewives and farmers [9]. To address this gap, the National AIDS Control Program (NACP) has initiated routine case-based surveillance [7] since 2020 among the reported new cases. Case-based surveillance was instituted to assess the detailed risk factors of new cases to understand the risk behaviors of the subpopulation groups contributing to the HIV epidemic. This is to allow prevention programs to target the high-risk sub-population groups for preventing new infections. However, those cases diagnosed before 2020 were not covered by the existing case-based surveillance; thus, their risk behaviors were not assessed.

Therefore, the detailed risk assessment of the diagnosed HIV cases in Bhutan is an opportunity to carry out the risk analysis to generate evidence of wherein, under what context, and in which population groups most new infections are occurring. The evidence collected will help to test the current assumption of Bhutan experiencing diffused and generalized HIV epidemic. The global evidence shows that such a pattern of HIV transmission is dynamic and changes over the period [10]. The periodic assessment and reassessment of risk behaviors of the HIV cases and how new infections are distributed among risk groups are critical for developing effective prevention strategies by overcoming the changing pattern of risk. Therefore, to determine the epidemiology of incident HIV infections in the country and assess the degree of alignment in between where the new cases of HIV are occurring, the risk assessment is important. This assessment aims to systematically understand the epidemic's nature by redefining the risk behaviors of HIV cases and its Mode of Transmission (MoT) for strategic investment in the overall national HIV response.

## 2. Methods

### 2.1. Study Design

This is a cross-sectional study using a qualitative method. The data collection was done from December 1 to 30 January 2022 in all the twenty districts of Bhutan. These districts were purposively selected to recruit the study population. The study protocol was reviewed and approved by the research ethics board of health. Confidentiality was explained, and informed consent was taken for the interview. The records were kept in locked cabinets of the Ministry of Health.

### 2.2. Study Population

This study included people living with HIV (PLHIV) in 20 Dzongkhags (Districts) of Bhutan diagnosed between 1993 and 2021 and who are currently living, born Bhutanese, who are ≥18 years, either on treatment or treatment naïve. In addition, all MTCT cases were also included directly from the existing data record to account for the MTCT route of transmission. The study has excluded those PLHIV who are sick, emotionally and mentally unstable including minors born from HIV-positive mothers.

### 2.3. Sampling Method and Sample Size

A convenient nonrandom sampling method was used to recruit the study population. A total of 404 PLHIV were recruited for the study which included 350 cases diagnosed from 1993 to 2019 that are not captured by current routine case-based surveillance since it came into effect only in 2020, and 54 cases diagnosed between 2020 and 2021, captured by the current surveillance. The overall sample size also included 39 MTCT cases from 1993 to 2021 for the MoT analysis. The MTCT cases were not included in the detailed risk assessment.

### 2.4. Recruitment of the Respondent

Owing to the sensitivity of the study population, the researchers coordinated with the respective HIV counsellors from the voluntary counselling and testing (VCT) centers across the country and health information and service centers (HISC) located in six major urban towns of Bhutan. The counsellor also works as an HIV case manager and has good professional linkages with the target population. Therefore, we have recruited the HIV Counselors as assistant researchers to administer the risk assessment survey for primary data collection, while the secondary data needed for analysis are sourced directly from the main database maintained at the NACP.

### 2.5. Data Collection Tools

We used the case-based surveillance questionnaires and guidelines to ascertain unbiased responses from the target population. Some of the key variables included in the case-based surveillance questionnaires are sociodemographic characteristics, risk factors for HIV (History of **s**exual contact with the male, female, transgender, bisexual, or female sex workers including transactional sex), injecting drug use, blood transfusion, HIV testing result, the reason for testing, testing results of hepatitis (B and C), tuberculosis, and then information on ART treatment and index testing for partner notification. However, this study analyzed only the sociodemographic characteristics, the reason for testing and risk factors related to HIV.

### 2.6. Data Processing and Analysis

Data were computed using EpiData Analysis software (version v3.0.0.1) and Microsoft Excel. The national risk assessment algorithm was used to analyze HIV cases involving a two-step process. The national risk assessment algorithm has been adapted and modified from the UNAIDS manual for modelling the expected short-term distribution of incidence of HIV infection by exposure group [11] and CDC risk ascertainment guidelines [12,13]. The risk analysis algorithm uses the following list of acronyms to define the risk factors:

1. HETHIV-Heterosexual contact with an HIV-infected person
2. HETHOM-Heterosexual contact with a bisexual person
3. HETIDU-Heterosexual contact with a person who injects drugs
4. HETF-Heterosexual contact reported by a female
5. HETM-Heterosexual contact reported by a male
6. HETSW-Heterosexual contact with a sex worker or client of a sex worker
7. HOM-Homosexual contact
8. HRH-High-risk heterosexual, includes HETHIV, SW, HETIDU, HETHOM, HETSW
9. IDU-Injecting drug use-related risk
10. NOS-Nosocomial risk
11. SP-Skin-penetration- related risk
12. SW-Sex-work-related risk

#### 2.6.1. Step 1. Analysis of Existing Risk Behavior and Risk Count Calculation

As indicated in Table 1, step one of the algorithms was divided into two parts: general existing risk assessment and then risk attribution analysis. All the risk variables from 3a.1 to 3a.7 and then from 3b.1 to 3b.5 were analyzed against the client's gender or sex to understand the overall risk of the respondents. The risk attribution was calculated using the counts and proportion against each risk behavior considering the overlapping sexual contacts among individual cases. Accordingly, each existing risk behavior ranging from 3a.1 to 3a.7 and then heterosexual risk factors ranging from 3b.1 to 3b.5 of the case-based surveillance were assessed, respectively. A possible hierarchy of risk behaviors as per the national risk analysis algorithm was stated as IDU>MSM>HRH *(HETHIV>SW>HETIDU>HETHOM>HETSW)*>SP>NOS>HETF>HETM).

#### 2.6.2. Step 2. Assess How Many Risk Factors Were Reported

In step two, analysis was carried out to understand the MoT by mapping the calculated risk attribution into the respective MoT defined by the national algorithm as indicated below. The MoT is assigned based on the reported risk behavior of the highest hierarchy with the following categorization:

(i) If none: MoT is unknown;
(ii) If one risk factor was reported: MoT = Risk factor:
    IDU, NOS, SP=Parenteral MoT;
    SW, HETF, HETM, HETHOM, HETSW, HETIDU, HETHIV=Heterosexual MoT;
    HOM=Homosexual MoT

If more than one risk factor was reported, MoT should take the value of the risk factor associated with a greater probability of transmission per act and a higher level of prevalence in respective key populations based on the risk hierarchy defined above.

## 3. Results

### 3.1. Sociodemographic Characteristics

Of the 404 cases whose risk profiles were analyzed, 48.26% (*n* = 195) were males, 51.23% (*n* = 207) were females, and 0.49% (*n* = 2) were transmen. The median age of the study population was 41 years with a minimum of 18 and a maximum of 77 years. About 75.49% (*n* = 304) of the reported cases belong to the age group of 25–49, and 13.36% (*n* = 54) were between the ages of 18 and 24 years, and 10.89% (*n* = 44) were above 50 years at the time of diagnosis. In terms of marital status, 66% (*n* = 267) of the reported cases were married, 17% (*n* = 69) were divorced, 9% (*n* = 36) were widowed, and 8% (*n* = 32) were single. When assessing their educational background, about 38% had no education, 30% were with middle secondary, 23% had primary education, and 3% were tertiary, while monastic education was just 5%.

### 3.2. Reason for HIV Testing

A separate analysis was carried out using the mode of diagnosis variables. Of the 404 cases, 120 (29.70%) were diagnosed through VCT, 113 (27.97%) from contact tracing, 83 (20.54%) through medical screening, 48 (11.88%) from antenatal clinics (ANC), 24 (5.94%) screened as blood donors, 7 (1.10%) patients with Tuberculosis (TB), 8 (1.1%) from sexually transmitted infections (STI) clinics, and 2 (0.49%) with clinical symptoms.

### 3.3. Analysis of the Risk Attribution due to Heterosexual Risk Behaviors of the Reported Cases

Taking into account that the majority of reported cases were heterosexual contacts, a detailed analysis of the risk attribution was carried out. As shown in Table 2, about 86.4% (*n* = 349) of the respondents had shown heterosexual contacts with sex workers or clients of sex workers (HETSW). 28.2% (*n* = 114) of the respondent revealed heterosexual contact with an HIV-infected person (HETHIV). The risk attribution of heterosexual contact with a bisexual male (HETHOM) and heterosexual contact with a person who injects drugs (HETIDU) constituted 1.0% of each.

### 3.4. Mode of Transmission Analysis Based on the Risk Attributions

As per the case-based surveillance risk analysis algorithm, the different risk attributions were mapped into different MoT categorically (Table 3). About 81.94% of the respondents have acquired the infection through high-risk heterosexual (HRH) followed by mother-to-child transmission with 9%, a low-risk heterosexual transmission that is heterosexual contact by males (HETM) and heterosexual contact by females (HETF) with 6.1% and 1.58% through a parenteral transmission consisting of IDU, nosocomial infections, and intentional skin penetration like tattooing etc., and 1.35% contributed via homosexuality. The analysis indicated five main MoT occurring among the reported HIV cases in Bhutan. Among them, the HRH consisting of HETHIV, SW, HETIDU, HETHOM, and HETSW is dominating the MoT.

### 3.5. Distribution of Heterosexual Mode of Transmission

**Figure 1:**
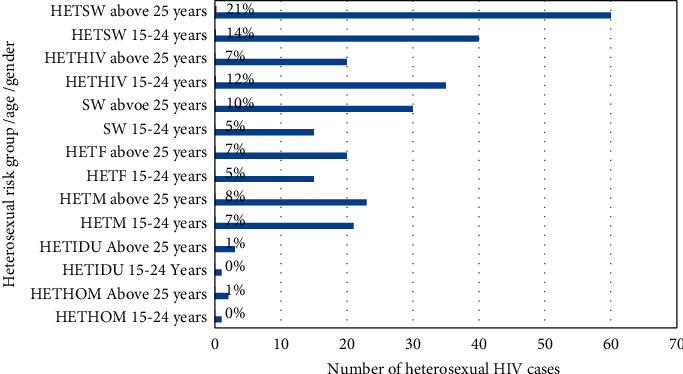
Distribution of heterosexual mode of transmission by age, gender, and risk group among the diagnosed HIV cases from 1993 to 2020, Bhutan.

Figure 1 shows the percentages, by age group and gender, of all new infections among adults from 1993 to 2020 that are attributable to heterosexual multiple partnerships with the sex workers or clients of sex workers and their spouses. Results were disaggregated for age groups 15–24 and ≥25 years old. Among the HETSW, majority (21%) were ≥25 years, whereas, for HETHIV, higher proportion (12%) is between the ages of 15–24 years. The remaining heterosexual risk group/ages and gender have almost equal proportions. The highest HIV transmission has occurred through HETSW (35%), HETHIV (19%), and then sex work (15%).

Out of 390 respondents who have engaged in heterosexual contact, 46.67% had acquired the infection through heterosexual contact with sex workers/clients of sex workers (HETSW), 31.70% from heterosexual contact with HIV-infected persons (HETHIV), and 14.36% from sex work. Only a negligible percentage of respondents had acquired HIV through heterosexual contact with a person injecting drug use (HETIDU) as reported by both male and female (HETM/F) respondents.

### 3.6. Redefined Mode of Transmission

**Figure 2:**
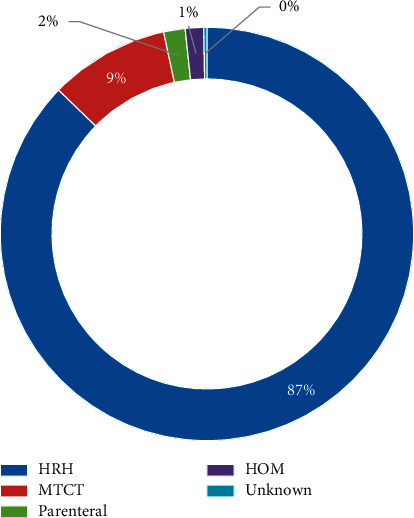
Redefined mode of transmission of reported HIV cases from 1993 to 2020 in Bhutan.

Based on the analysis of the different risk attributions versus the MoT shown in Figure 2, the MoT is redefined broadly into four main risk attributions. The predominate MoT is through the high-risk heterosexual (HETHIV, SW, HETIDU, HETHOM, and HETSW) which constituted 81.94% (*n* = 363) and followed by MTCT with 9% (*n* = 39), thus making it the second highest MoT. The parenteral (IDUs, NOS, and SP) and homosexuality, respectively, attributed to 2% each.

## 4. Discussion

The detailed risk analysis of the respondents overwhelmed the current perception of Bhutan experiencing a diffused and generalized HIV epidemic [14]. It is evident from this study that the cumulative proportion of HIV infection among pregnant mothers is only 11.88% of the reported cases, indicating an infection occurring among the high-risk groups instead of the general population. This is true from the global literature where it mentioned that in any kind of generalized HIV epidemic more cases are likely to come from the ANC clinics because pregnant women are being used as a proxy for the general population [15, 16]. As per the UNAIDS definition, the generalized epidemic is those where HIV prevalence exceeds 1% in the general population, which is often represented by antenatal clinic (ANC) attendees [17]. However, in Bhutan's case, the overall adult (>15 years) HIV prevalence is below 0.23% [1] coupled with a low prevalence of ANC cases as discussed above. Furthermore, the analysis of registered HIV cases in Bhutan showed a decreasing female-to-male ratio in the last 5 years in comparison to the previous 5 years from 1.02 : 1 to 0.93 : 1, indicating a potential sign of other risky behaviors emerging [18]. As per the evidence from the African countries experiencing the generalized HIV epidemic, the HIV infection among heterosexual contacts is dominated by the female over the male. This is evident as the empirical estimates of the gender ratio of infections in African populations with an HIV prevalence level above 1% range from 1.3 : 1 in Zambia to 2.21 in the Ivory Coast [19]. Further, the UNAIDS epidemic report also suggests that about 60% of adults living with HIV in sub-Saharan Africa are women, and that corresponds to a female-to-male infection ratio of 1.48 [20]. This shows that the likelihood of Bhutan experiencing a subpopulation concentrated HIV epidemic is high although more empirical evidence is needed.

Interestingly, this study discovered that the majority of the infection occurs through high-risk heterosexuals contact with sex workers and clients of sex workers followed by HIV persons. This exhibits the likelihood of the HIV infection coming from these subpopulation groups which remained a neglected issue to date. In general, the domination of MoT through high-risk heterosexuals indicates the emergence of a concentrated HIV epidemic among subpopulation groups like female sex workers. The situation becomes, even more, riskier as the majority of reported cases have acquired the infection through heterosexual contact with HIV-positive persons. This demonstrates that HIV-infected persons have either transmitted the virus intentionally to their spouses or sexual partners and some through ignorance because of their unknown status mainly due to late diagnosis. The late diagnosis of HIV cases in Bhutan is evident from the recent findings where the mean time interval between the initial HIV infection and the first diagnosis was 4 years with the majority of them infected between 5 and 8 years [21].

As exhibited in this study, the heterosexual contact with a female sex worker was consistent with the results of several past national studies where the prevalence of female sex workers and high multiple sexual relationships with low condom use were reported [5, 22, 23]. The 2019 population size estimation (PSE) study also depicted higher sexual partners among women acknowledging sex work, with a mean of 5.8 and 4.5 for female sex workers recruited outside of venues and at venues, respectively, as compared to 1.3 for high-risk women not engaging in sex work. Consistent condom use was low across all women and partner types, although being higher with paying partners [5]. Further, the recent sentinel surveillance among the key population in Bhutan also showed 97% of high-risk women exchanging sex for money and also reported the highest level of unprotected sex in the last 30 days (76%) [24].

Although the MoT through homosexuality is low among the current reported cases, it is hard to conclude that no transmission is happening through homosexual behavior among the Bhutanese population. This is clear from the increasing network of MSM and transgender persons coupled with the growing high-risk behaviors of these communities. For example, the recent sentinel surveillance found a majority of transgender women (59%) had engaged in commercial sex work with an average of 5.4 partners in the last 30 days and 1.8 different sexual partners for transgender women and MSM, respectively. The past evidence also showed the existence of multiple sexual relationships among the MSM consisting of both male and female partners [25]. Further, two new HIV infections among the MSM communities were reported very recently [6]. This shows that the infection is slowly picking up among MSM communities demanding robust routine surveillance and scaling up of community-based testing for early diagnosis.

The other reason attributed to low HIV prevalence among MSM could be the existing stigma and discrimination barring them from revealing their true sexual orientation. This is evident from the PSE study 2019 where only 4.8% mentioned that their MSM status was known to many people and reported that social stigma was highest among the MSM and transgender persons [16]. The global evidence also showed that many MSM persons have experienced homophobic stigma, discrimination, and violence, thereby resulting in hiding their identity and sexual orientation. Some findings also revealed that MSM and transgender persons fear a negative reaction from healthcare workers [26–28]. As a result, MSM and transgender persons are less likely to access HIV and other services by revealing their true information during the risk assessment at the time of treatment initiation. Therefore, the growing risk behaviours coupled with prevailing self and social stigma among MSM and transgender persons can be one of the potential risk factors for HIV/AIDS in the future besides the risk behavior associated with female sex workers and their clients.

The parenteral transmission contributed to about 2% of the overall HIV infection in the country mainly through the IDU, nosocomial infections, and skin penetration. However, it is important to note that parenteral transmission can be mainly through IDUs or intentional skin penetration instead of nosocomial infection. This is because all blood and blood products are well screened for any infectious diseases before it is being donated or transplanted to another person based on the existing national policy [29]. The existing transfusion-transmitted infection (TTI) guidelines recommend screening of all blood and blood products for a virus, parasite, or another potential pathogen that can be transmitted in donated blood and blood products. The global evidence shows that 10% of HIV and AIDS cases worldwide are attributed to IDUs [30]. The IDUs in different regions contributing to HIV epidemics are well documented, unlike in Bhutan [31,32].

Heterosexual contact with HIV-infected person, sex workers and their clients, IDUs, and homosexuals were found to be the main MoT among the reported HIV cases in Bhutan. As a result, the preexisting assumption of infection coming from the general population seems to be not true. Therefore, it was found critical for the country to redefine the existing MoT and establish evidence-based risk behaviors in the reported cases. Accordingly, the existing occupational-based risk behavior has to be replaced with the epidemiologically defined risk behaviors as revealed in this study. However, it is vital to carry out risk analysis periodically as a part of the routine case-based surveillance to understand the changing risk behavior of the new cases. One important observation made from this study was that the current case-based surveillance form does not segregate sex workers and clients of sex workers to understand whether clients of sex workers are from a stable relationship or not. In addition, the case-based surveillance form also does not capture the information on the HIV status of the spouse to determine the heterosexual contact with his or her spouse at the time of risk assessment. This is important because many HIV-seropositive individuals who are aware of their infection and who are engaged in high-risk activity may also pose a significant risk for transmission in the community [33]. This is evident from this study where 28.2% of the reported cases got infected through heterosexual contact with HIV-infected persons.

This study is also subject to some information bias. However, we tried to overcome these limitations by using experienced HIV counsellors who are also routine caregivers of HIV patients. The field investigators are also trained well on the survey tools including the guidelines for completing risk assessment to ensure they deduce down to evident risk. What matters is the revelation of true information but that is likely to be resolved as they are being approached by their most trusted caregiver.

## 5. Conclusions

This study depicted that the dominant mode of transmission among the reported HIV cases in Bhutan is contributed by high-risk heterosexuals such as heterosexual contact with sex workers and their clients including HIV-positive persons. Therefore, the existing assumption of diffused and generalized HIV epidemic in Bhutan seemed not true, and the epidemic was more likely to be concentrated among high-risk subpopulation groups like female sex workers and their clients. Therefore, the need to focus on HIV prevention activities including community-led testing among female sex workers and their clients instead of the general population is critical for early diagnosis and treatment to bridge the current 39% case detection gap of the estimated 1300 HIV cases.

## Figures and Tables

**Table 1:** Assessment of all the existing risk behaviours based on the following national algorithm.

Question	Risk factor attribution
3a. 1 Did you ever have sexual contact with a male?	If male or transwoman and yes – HOM
	If female and yes - HETF
	Otherwise - unknown

3a. 2 Did you ever have sexual contact with a female?	If male or transwoman and yes - HETM
	Otherwise – Unknown

3a. 3 Did you ever have sexual contact with a transgender person?	If male or transwoman and yes - HOM
	Otherwise – Unknown

3a. 4 Did you ever inject any drugs not prescribed by a physician?	If yes - IDU
	Otherwise – Unknown

3a. 5 Did you ever receive blood or blood products transfusion or transplant?	If yes - NOS
	Otherwise - unknown

3a. 6 Did you ever have any invasive surgical procedures?	If yes - NOS
	Otherwise - unknown

3a. 7 Did you ever have intentional skin penetration, e.g., tattoo, scarring, piercing?	If yes - SP
	Otherwise - unknown
If heterosexual sex was reported:	

3b. 1 Did you ever have heterosexual contact with an HIV-infected person?	If yes – HETHIV
	Otherwise - unknown

3b. 2 Did you ever provide sexual services for money, gifts, or any other kind of remuneration?	If yes – SW
	Otherwise - unknown

3b. 3 Did you ever have heterosexual contact with a person who injects drugs?	If yes – HETIDU
	Otherwise - unknown

3b. 4 Did you ever have heterosexual contact with a bisexual male?	If yes – HETHOM
	Otherwise - unknown

3b. 5 Did you ever have heterosexual contact with a sex worker or client of a sex worker?	If yes – HETSW
	Otherwise - unknown
Section 7. Viral hepatitis testing	If HCV- is positive – IDU (given the strong correlation between HCV and IDU, the presence of anti-HCV antibodies in many contexts is considered a marker of IDU-related transmission)

**Table 2:** Risk attribution based on the heterosexual-related risk behavior of the diagnosed HIV cases from 1993 to 2021, Bhutan.

Q. No	Heterosexual risk factors (*N* = 404)	Yes		Risk attribution
		*n*	Percentage (%)	
3b.1	Ever having heterosexual contact with an HIV-infected person.	114	28.2	HETHIV
3b.2	Ever providing sexual services for money, gifts, or any other kind of remuneration.	54	13.4	SW
3b.3	Ever having heterosexual contact with a person who injects drugs.	4	1.0	HETIDU
3b.4	Ever having heterosexual contact with a bisexual male.	4	1.0	HETHOM
3b.5	Ever having heterosexual contact with a sex worker or client of a sex worker.	349	86.4	HETSW
	Total risk count	525	130	

*Note.* The percentage does not add up to 100 because of overlapping risk factors of the individual cases.

**Table 3:** Mapping of risk factors against the Mode of Transmission.

Risk factor	*N*	Percent (%)	MoT
Injecting drug use (IDU)	3	0.68	Parenteral
Nosocomial (NOS)	2	0.45	
Skin-penetration (SP)	2	0.45	
Subtotal	**7**	**1.58**	
Heterosexual contact reported by a female (HETF)	19	4.29	Heterosexual
Heterosexual contact reported by males (HETM)	8	1.81	
Subtotal	**27**	**6.09**	
Heterosexual contact with HIV-infected person (HETHIV)^*∗∗*^	124	27.99	High-risk heterosexual (HRH)
Sex workers (SW)^*∗∗*^	56	12.64	
Heterosexual contact with HIV with a person injecting drug use (HETIDU)^*∗∗*^	1	0.23	
injects	0	0.00	
Heterosexual contact with sex workers/clients of sex workers (HETSW)^*∗∗*^	182	41.08	
Subtotal	**363**	**81.94**	
Homosexual contacts (HOM)	6	1.35	Homosexual
Subtotal	**6**	**1.35**	
Mother to child transmission (MTCT)	39	8.80	MTCT
Total	**39**	**8.80**	
Unknown risk	1	0.23	Unknown
Subtotal	**1**	**0.23**	
Overall total risk count	**443**	**100**	

## Data Availability

The readers can access the data supporting the conclusions of the study on a case-by-case basis by emailing directly to the corresponding author.
